# Trajectory Patterns of Macronutrient Intake and Their Associations with Obesity, Diabetes, and All-Cause Mortality: A Longitudinal Analysis over 25 Years

**DOI:** 10.3390/nu16152567

**Published:** 2024-08-05

**Authors:** Jingxian Huang, Rong Rong, Zheng Feei Ma, Ying Chen

**Affiliations:** 1 Department of Biological Sciences, School of Science, Xi’an Jiaotong-Liverpool University, Suzhou 215123, China; jingxian.huang22@imperial.ac.uk; 2 Department of Epidemiology and Biostatistics, School of Public Health, Faculty of Medicine, Imperial College London, London SW7 2AZ, UK; 3 Department of Health Policy and Management, School of Public Health and Tropical Medicine, Tulane University, New Orleans, LA 70112, USA; rrong@tulane.edu; 4 Centre for Public Health and Wellbeing, School of Health and Social Wellbeing, College of Health, Science and Society, University of the West of England, Bristol BS16 1QY, UK; zhengfeei.ma@xjtlu.edu.cn; 5 Wisdom Lake Academy of Pharmacy, Xi’an Jiaotong-Liverpool University, Suzhou 215123, China

**Keywords:** nutrition transition, chronic diseases, longitudinal dietary pattern, macronutrients, China health, diabetes, obesity

## Abstract

Over the past decades, China has been undergoing rapid economic growth, which may have significantly influenced the dietary patterns and health status of the Chinese population. Our study aimed to assess the associations of potential macronutrient trajectory patterns with chronic diseases and all-cause mortality using the latent class trajectory model (LCTM) and the longitudinal data of the China Health and Nutrition Survey obtained between 1991 and 2015. A 24-hour diet recall was used to assess the dietary intake. The Poisson regression model was employed to investigate the correlations between trajectory patterns and chronic diseases and all-cause mortality. A total of 8115 participants were included in the final analysis. We explored four and three trajectory patterns for male and female populations, respectively. We found that a decreasing very high-carbohydrate trajectory together with a U-shape protein trajectory was associated with a higher risk of diabetes in the male population (odds ratio (OR): 2.23; 95% confidence interval (CI): 1.31–3.77). A similar pattern for moderate protein intake was also associated with the risk of diabetes in the female population (OR: 1.82; 95% CI: 1.18–2.79). In addition, we show that a decreasing low-carbohydrate trajectory and an increasing high-fat trajectory were associated with a lower risk of all-cause mortality (OR: 0.76; 95% CI: 0.60–0.96) and a higher risk of obesity (OR: 1.24; 95% CI: 1.05–1.47) in males. Our results shed light on some salient nutritional problems in China, particularly the dual challenges of undernutrition and overnutrition.

## 1. Introduction

Since China’s reform and opening-up in 1978, China’s economy has experienced a rapid growth with the average annual growth rate of the gross domestic product (GDP) reaching 9.4% from 1979 to 2018 [1]. The burgeoning economy, which has been the world’s second largest economy in terms of GDP since 2010 [2] and the largest in terms of purchasing power-adjusted GDP since 2014 [3], catalyzed the country’s fast urbanization and also the emergence and expansion of a modern food system. Consequently, unprecedented and dramatic changes have occurred in China’s food consumption patterns and eating and cooking behaviors, causing profound effects on the various macro- and micro-nutrient intakes of the population [4,5]. Studies have uncovered a transition in the Chinese dietary patterns from a traditional diet toward a Western and modern style diet, with a decline in the consumption of cereals and vegetables but an increase in fat and animal-based and processed foods [6,7]. The shifting dietary trends have also been identified in several other studies on the same population within a similar time period [8,9,10]. More importantly, more and more research has highlighted the significant association between the dietary transition and the increasing prevalence of a number of non-communicable diseases, including obesity, diabetes, cardiovascular diseases and certain types of cancer [11,12,13,14].

As all diets contain a mixture of the three macronutrients, i.e., carbohydrate, fat, and protein, one of the approaches that has been frequently applied to explain diet composition, its variations, and potential health implications is to examine macronutrient intake [15]. Macronutrients not only serve as the exclusive source of energy for the human body, but also play crucial roles in mediating and maintaining the health of individuals [16,17,18]. Therefore, the changes in the macronutrient consumption at the population level, which are typical during the rapid economic development and urbanization of a society, could impact the population’s health [19,20]. However, there is limited evidence revealing the trends and patterns of the population’s macronutrient intake over the course of a drastic societal transition.

With an emphasis on dietary energy intake, previous studies often involved calculating the proportion of energy from each macronutrient of the total dietary energy intake, i.e., the carbohydrate-to-energy ratio (CER), fat-to-energy ratio (FER), and protein-to-energy ratio (PER) [19,21]. Despite the intensive efforts to elaborate the health effects of various dietary patterns with differing levels of macronutrients, mostly represented by CER and FER, inconsistent and even controversial conclusions were drawn [19,22,23,24]. This may be partly due to the different methodologies used to measure CER and FER, of which the cumulative intake method is suggested to have more advantages than the single-time point method in reducing measurement error and reflecting the real-world dietary intake [25,26]. Nevertheless, both methods are deficient in capturing the temporal trend in macronutrient intake. Moreover, few studies adopted the absolute energy intake of macronutrients, rather than the energy ratio, to assess the health impacts of dietary patterns, even though both the energy and non-energy attributes of macronutrients could be related to certain health risks. Additionally, investigations into the variations in absolute macronutrient intake at the population level and their associations with long-term health outcomes based on nationwide longitudinal data are rare.

The primary objective of this study is to detect the longitudinal trajectories of dietary nutrition transition (i.e., the intake of carbohydrate, fat, and protein) using a latent class trajectory model (LCTM) among the Chinese population from 1991 to 2011, a 20-year period when rapid economic growth and urbanization were accompanied by substantial changes in the citizen’s dietary patterns. Furthermore, we aim to identify distinguishing features by combining the trajectories of each of the three macronutrients using conventional latent class analysis (i.e., trajectory patterns) for the male and female populations, separately. The potential associations of the trajectory groups with selected non-communicable diseases and all-cause mortality are also investigated.

## 2. Materials and Methods

### 2.1. Study Population

The China Health and Nutrition Survey (CHNS) is an ongoing nationwide longitudinal survey that was designed to investigate nutritional transitions and their interactions with socio-economic factors in the Chinese population [27]. A multi-stage random cluster approach was used for survey sampling [28]. The CHNS includes ten completed waves in 1989, 1991, 1993, 1997, 2000, 2004, 2006, 2009, 2011, and 2015, with over 7200 households and 30,000 participants [28]. In this study, we used the wave of 1991 as the cohort baseline and included participants who provided dietary information in five or more waves of the surveys between 1991 and 2011. The final study population included 8115 participants with 52,486 responses; thus, on average, 6.5 sets of dietary information were obtained from each participant over eight waves. Participants were followed up until the last wave conducted in 2015. Participants were from nine provinces (Liaoning, Heilongjiang, Jiangsu, Shandong, Henan, Hubei, Hunan, Guangxi, and Guizhou) located throughout China.

### 2.2. Measures of Dietary Intake

Dietary data were measured by trained interviewers for three consecutive days over one week [27]. The changes in food inventory were examined from the beginning to the end of each consecutive day. All purchased or home-produced foods, snacks, and preparation waste were recorded and weighed. At the individual level, a 24-hour recall method was used for daily food consumption. In addition, both outdoor and at-home food consumption data were recorded with the aid of food pictures and models. The quality of the dietary assessment was carefully cross-checked [27]. Macronutrient (carbohydrate, fat, and protein) intake from the recorded food items for each participant was calculated according to the Chinese Food Composition Table [29]. The three-day average macronutrient intake was reported as g/day. On the basis of conversion factors between macronutrients and kilocalories (carbohydrate and protein, 4 kcal/g; fat, 9 kcal/g), the three-day average energy intake was calculated as kcal/day. CER, FER, and PER were calculated as the energy from a particular source (macronutrient/total, in %). Participants with implausible energy intake (<500 kcal/day or >6000 kcal/day) were entered as ‘NAs’ for data imputation.

### 2.3. Measures of Health Outcomes

The outcome variables were all-cause mortality, cancer, stroke, myocardial infarction, hypertension, diabetes, and obesity. All-cause mortality was recorded according to the CHNS document regarding the death of participants. Information on cancer, stroke, myocardial infarction, and diabetes was based on self-reported diagnosis history or receiving relevant treatments. Hypertension status was determined based on self-reported diagnosis history, taking anti-hypertension drugs, systolic blood pressure ≥140 mm Hg, or diastolic blood pressure ≥90 mm Hg. Obesity status was defined when an individual’s body mass index (BMI) was ≥28 kg/m^2^ [30]. BMI was calculated based on an individual’s height (without shoes, closest to 0.1 cm) and weight (closest to 0.1 kg). Diseases identified at the baseline (1991) were regarded as the pre-existing conditions, while those identified in the subsequent survey waves (1993–2015) were considered newly developed onsets (i.e., incident cases).

### 2.4. Covariates

Important sociodemographic and lifestyle characteristics were obtained for each participant, including age, BMI, geographical region, residential area (rural vs. urban), education level, household income, marital status, and status of alcohol and smoking consumption. The geographical region was reported as a binary term of individual provinces allocated to the South and North based on the Qinling Mountain–Huai River Boundary. Education level was categorized into three levels: low (primary school and below), medium (junior high school), and high (senior high school, technical school, and above). Per capita household income was divided into tertiles as low, medium, and high. Smoking status (cigarette and pipe combined) was identified as never, former, or current smoking, whereas alcohol intake was recorded as never, less than one time per week, or one or more per week.

### 2.5. Statistical Analysis

The latent class trajectory model (LCTM) was used to identify macronutrient trajectories over time (latent class mixed models package in R Studio) [31]. We built the model as a function of time to describe the changes in macronutrient intake. We considered the quadratic terms of time for the model with fixed effects and random intercept and slope for the model with random effects. Additionally, we allowed the variance–covariance matrix to vary across different clusters. Our LCTM models were constructed for each macronutrient over 1991–2011 for both genders separately, and the optimal model (i.e., the optimal number of trajectories) was identified from the models with one to five trajectories [31,32]. We determined the optimal models based on the following criteria: a successfully converged model having the lowest Bayesian information criterion (BIC) and a model including at least 2.5% of the total sample of participants in each derived trajectory. We then adopted latent class analyses (in Latent GOLD version 4.5) to determine groups with similar compositions of macronutrient trajectories (i.e., trajectory patterns). Using the results obtained from the LCTM, each participant was assigned to a particular trajectory based on different macronutrients. We also evaluated the optimal models among the models with one to five trajectory patterns and selected the optimal model following the aforementioned criteria. Baseline characteristics across different trajectory patterns were described and compared. In comparison, we used analysis of variance for continuous data with a normal distribution and the Kruskal–Wallis test for data without normal distribution. Chi-squared tests were used to compare discontinuous data. A Poisson regression model with a robust estimation of variance was employed to investigate the relationships between macronutrient patterns and health outcomes. Patterns assigned to the largest proportion of the study population were taken as the referent. Participants with relevant pre-existing conditions were excluded from the regression models; thus, the study population for individual analyses of different outcomes may be different. The unadjusted model (Model 1) and the model with full confounding adjustment for age, geographic region, location, education level, household income, marital status, alcohol consumption, smoking status, BMI, CER, FER, and PER (Model 2) were reported. Effects of associations were measured using odds ratios (ORs) with 95% confidence intervals (CIs).

All statistical analyses were carried out in R Studio (version 1.4.17) if not stated otherwise. Particularly, the missing rate of the macronutrient data was 19.4%. Missing data were imputed using multivariate imputations with the chained equations package in R Studio [33]. The statistically significant threshold was set as a two-tailed *p*-value < 0.05.

## 3. Results

### 3.1. Trajectories of Macronutrient Intake

**Figure 1:**
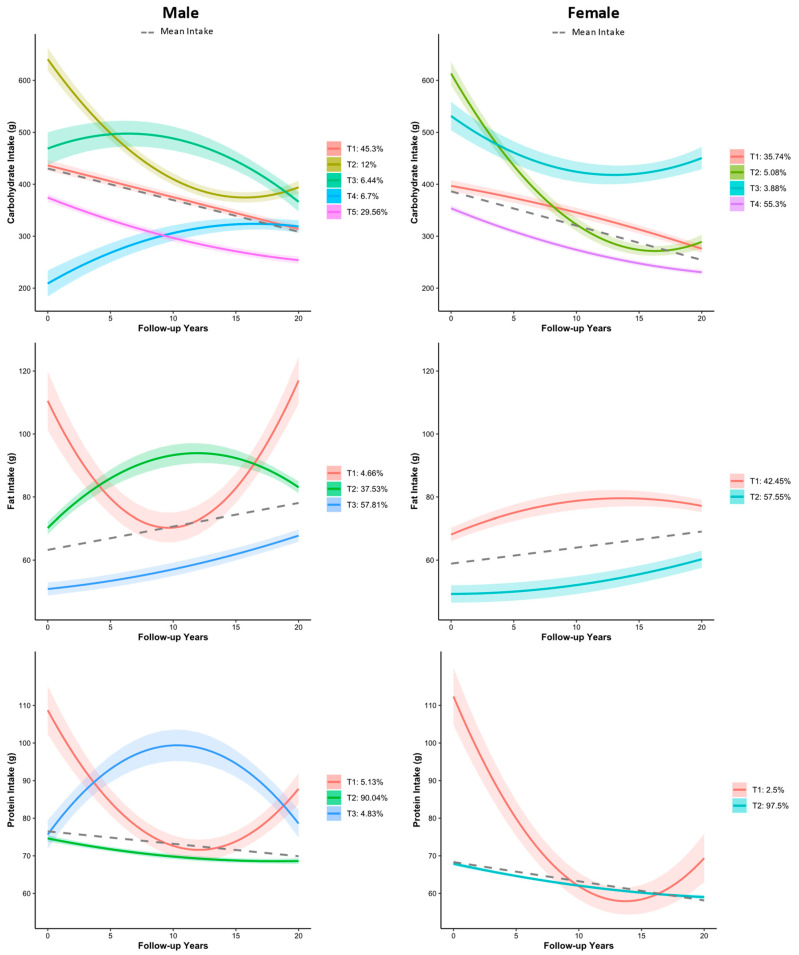
Trajectories of carbohydrate, fat, and protein among different genders from 1991–2011 based on the latent class trajectory model. Shades of the color indicate the 95% confidence intervals. The dash lines represent the trajectory of the mean intake across time.

Generally, over the years of 1991–2011, we observed a significant decline, a moderate increase, and a significant increase in the intakes of carbohydrate, protein, and fat, respectively, in both males and females of the studied population (Figure 1). The LCTM parameters for selecting the optimal models are shown in Supplementary Tables S1 and S2 for the male and female populations, respectively. The derived macronutrient trajectories for both the male and female populations from the LCTM are shown in Figure 1.

### 3.2. Trajectory Patterns

**Figure 2:**
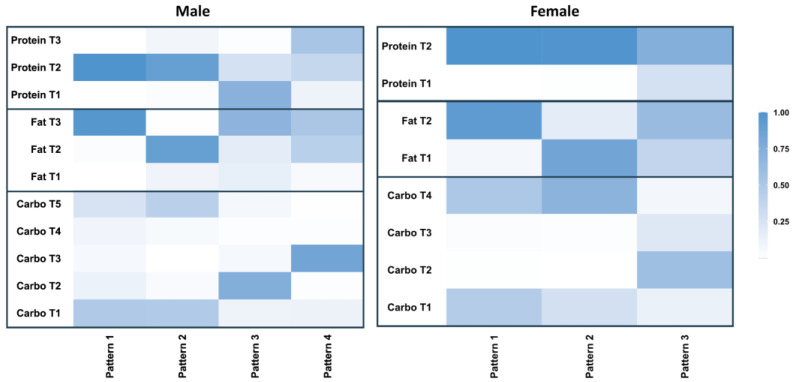
Heat maps for the compositions of different trajectory patterns among male and female populations.

**Figure 3:**
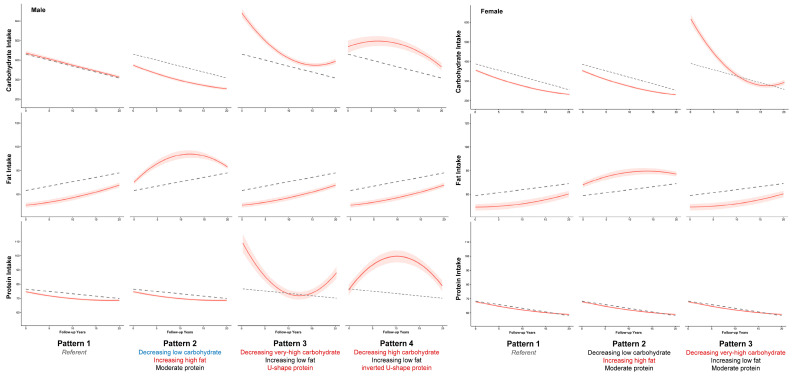
The most frequent macronutrient trajectories for each trajectory pattern in the male and female populations. The red lines represent the most frequent trajectories within each pattern; the dash lines represent the trajectory of the mean intake across time.

We identified four trajectory patterns for males and three trajectory patterns for females based on the selection of the optimal models (Supplementary Table S3). The compositions of each pattern are shown in Figure 2. We used the most frequent trajectories of carbohydrate, fat, and protein to describe each overall pattern, as illustrated in Figure 3. For example, the largest pattern in males (contained 54% of the male population) based on the most frequent trajectories of macronutrient intake was characterized as moderate carbohydrate (MC), increasing low fat (ILF), and moderate protein (MP) (pattern 1: MC&ILF&MP). Other patterns for the male population included pattern 2, which included 39.3% of males and was characterized as decreasing low carbohydrate (DLC), increasing high fat (IHF), and MP (DLC&IHF&MP); pattern 3, which contained 4.2% of males and was characterized as very high but drastically decreasing carbohydrate (DVHC), ILF, and U-shape protein (UP) (DVHC&ILF&UP); and pattern 4, which included 2.5% of males and was characterized as decreasing high carbohydrate (DHC), ILF, and inverted U-shape protein (IUP) (DHC&ILF&IUP).

As for the female population, the largest pattern, which included 53.7% of females, was characterized as DLC, ILF, and MP (pattern 1: DLC&ILF&MP). Other patterns included pattern 2, which included 40.5% of females and was characterized as DLC, IHF, and MP (DLC&IHF&MP), and pattern 3, which included 5.8% of females and was characterized as DVHC, ILF, and MP (DVHC&ILF&MP).

### 3.3. Population Characteristics by Trajectory Patterns

Baseline characteristics of the study population, stratified by the identified trajectory patterns, are shown in Table 1. Apart from alcohol intake and smoking status in the female population, other baseline characteristics were all significantly different across these trajectory patterns.

### 3.4. Associations between Trajectory Patterns and Health Outcomes

**Figure 4:**
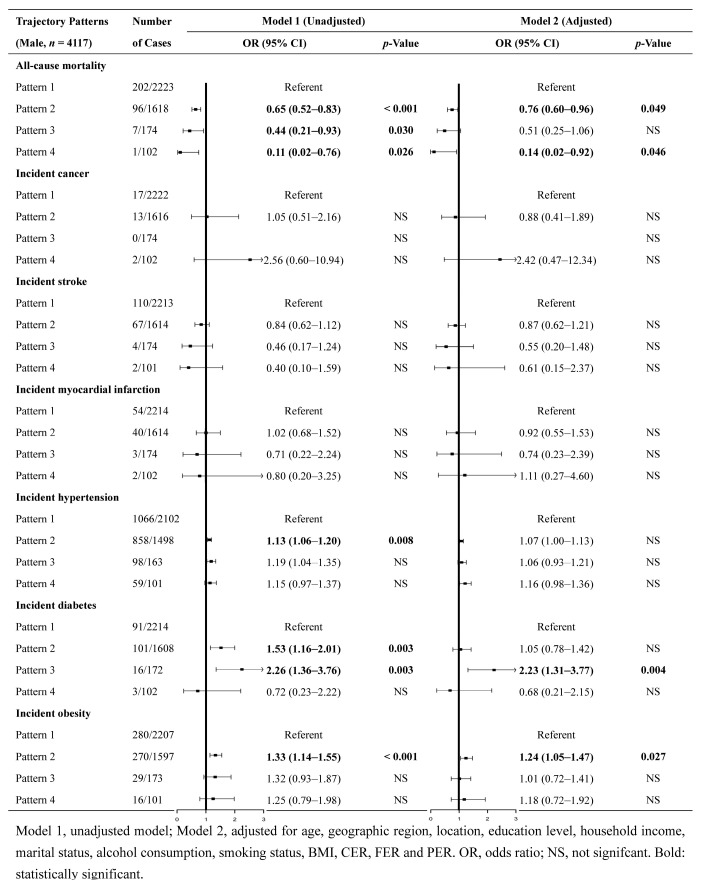
Associations between trajectory patterns and risks of diseases and all-cause mortality in the male population.

**Figure 5:**
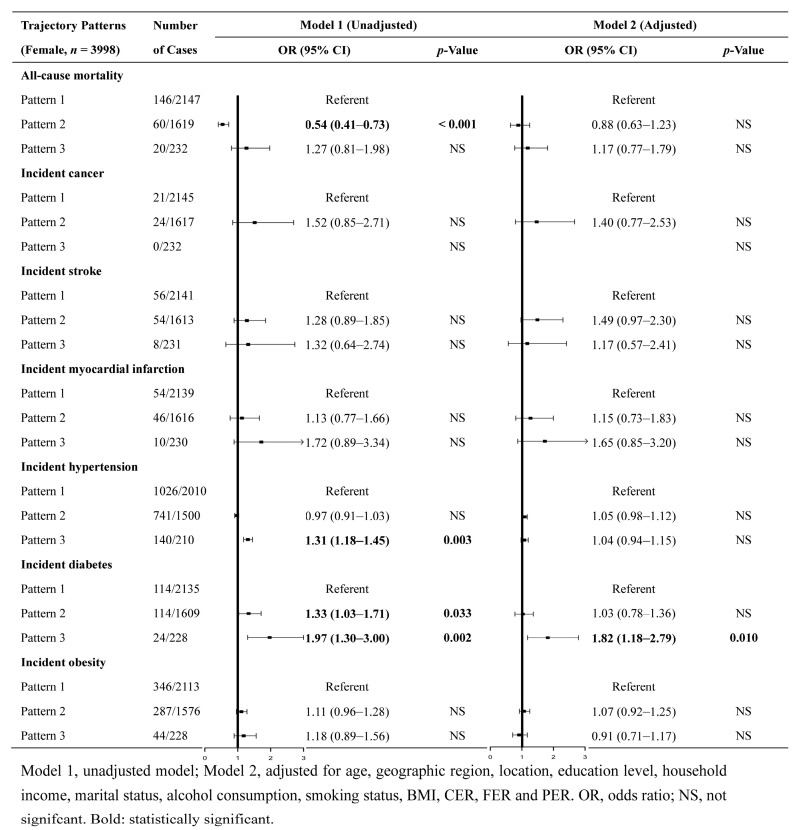
Associations between trajectory patterns and risks of diseases and all-cause mortality in the female population.

The associations between trajectory patterns and risks of diseases for the male and female populations are presented in Figure 4 and Figure 5, respectively. Among the male population, compared with the referent (pattern 1), pattern 2 (DLC&IHF&MP) was significantly associated with a lower risk of all-cause mortality (OR = 0.65, 95% CI = 0.52, 0.83) and a higher risk of hypertension (OR = 1.13, 95% CI = 1.06, 1.20), diabetes (OR = 1.53, 95% CI = 1.16, 2.01), and obesity (OR = 1.33, 95% CI = 1.14, 1.55). The associations with all-cause mortality (OR = 0.76, 95% CI = 0.60, 0.96) and obesity (OR = 1.24, 95% CI = 1.05, 1.47) were still statistically significant after adjusting for multiple covariates. Additionally, pattern 3 (DVHC&ILF&UP) was significantly associated with a higher risk of diabetes (OR = 2.23, 95% CI = 1.31, 3.77), and pattern 4 (DHC&ILF&IUP) was significantly associated with a lower risk of all-cause mortality (OR = 0.14, 95% CI = 0.02, 0.92). Both associations were maintained after adjusting for multiple covariates.

Among the female population, compared to the reference pattern (pattern 1), pattern 2 (DLC&IHF&MP) was significantly associated with a lower risk of all-cause mortality (OR = 0.54, 95% CI = 0.41, 0.73) and a higher risk of diabetes (OR = 1.33, 95% CI = 1.03, 1.71). However, none of these associations were deemed significant after the adjustment for multiple covariates. In addition, pattern 3 (DVHC&ILF&MP) was associated with a higher risk of hypertension (OR = 1.31, 95% CI = 1.18, 1.45) and diabetes (OR = 1.97, 95% CI = 1.30, 3.00) in the unadjusted model. However, only the association with diabetes (OR = 1.82, 95% CI = 1.18, 2.79) remained statistically significant after adjusting for multiple covariates.

## 4. Discussion

In this national prospective study, we used LCTM models and identified five trajectories of carbohydrate, three trajectories of fat, and three trajectories of protein among the male population. We also identified four trajectories of carbohydrate, two trajectories of fat, and two trajectories of protein among the female population. For both male and female populations, we observed a decline in total energy intake, carbohydrate intake, and protein intake (to a lesser extent) and an increase in fat intake. These findings were consistent with the general trend of the Chinese population between 1982 and 2012 reported elsewhere [34,35]. The decline in carbohydrate intake and increase in fat intake could be largely explained by the transition from a traditional diet (loaded with rice and flour) to a modern diet (loaded with fast food, cakes, and dairy products) and increased access to a variety of red meat [7,8]. The moderately declining trend in protein intake may be partly due to the ageing of our population. More heterogeneity in carbohydrate trajectories compared to other macronutrients and in the male population compared to the female population was captured using the LCTM. Certainly, the scale of carbohydrate intake allowed a greater variation compared to fat and protein intake. The disparities in these findings between males and females may be due to the dietary differences related to sexual orientation and gender expression [36].

We identified four trajectory patterns for the male population and three trajectory patterns for the female population based on the latent class analysis of the macronutrient trajectories. In the male population, we found that pattern 2 (DLC&IHF&MP) was associated with a lower risk of all-cause mortality. This pattern exhibited no significant abundance in carbohydrate and protein trajectories compared to the referent but was abundant in fat trajectory 2, which showed a high and increasing trend in fat intake. This finding was consistent with one large-scale prospective study that reported that the highest quintile of fat intake was associated with a lower risk of total mortality, regardless of the type of fat [22]. A recent meta-analysis on 19 prospective cohorts suggested a similar conclusion; however, they considered fat intake as FER [37]. Nevertheless, our finding built on existing evidence, suggested that a trajectory of increasing fat intake, together with a persistent low level of dietary carbohydrate, was protective against the risk of all-cause mortality. Our results also indicated that this pattern was associated with an increased risk of obesity. This is rather obvious as epidemiological evidence had linked fat intake and FER with anthropometric measures and obesity risk [38]. A recent study adopting a similar approach to our approach also suggested a positive correlation between FER and obesity [26].

Our results show that male pattern 3 (DVHC&ILF&UP) was associated with a higher risk of diabetes. This pattern uniquely consisted of DVHC and UP, compared to other patterns. Growing evidence has demonstrated that high consumption of dietary carbohydrates, in particular those with a high glycemic index, may have negative implications on glucose metabolism [39,40]. Indeed, a nonlinear association was suggested to exist between carbohydrate intake and diabetes risk, whereas too low or too high CER may both increase the risk [41]. In addition, carbohydrate quality was suggested to be an important mediator in this relationship [42]. On the other hand, high protein intake, in particular animal-based proteins, was reported to be a risk factor for type-2 diabetes (T2D) in diverse populations [43,44,45]. One possible underlying mechanism may be that elevated levels of circulating branched-chain amino acids (BCAAs) resulting from high dietary protein intake are implicated in the etiology of T2D through impaired BCAA metabolism [46]. Our findings may suggest that a high-carbohydrate and high-protein diet was associated with an increased risk of diabetes, while the transition to a lower level of intake was not effective enough to lower the risk. Further research is warranted to verify if the quality of carbohydrates and proteins would be the missing piece.

We also found that male pattern 4 (DHC&ILF&IUP) was a stronger protective pattern against all-cause mortality compared to the protective effect of male pattern 2. The most frequent carbohydrate trajectory in this pattern was DHC, which was inconsistent with several other studies that suggested a positive correlation between carbohydrate intake and all-cause mortality [19,26]. In addition, the IUP trajectory was abundant in this pattern, which demonstrated mild protein intake at an early stage, increasing to a higher level at the halfway point, and dropping to a mild level at the end. Prospective studies have shown a U-shape correlation between PER and all-cause mortality, which was mainly driven by animal-based proteins [47,48]. Nevertheless, only a small proportion of participants exhibited this pattern, and the number of cases was relatively small; therefore, this finding should be treated with caution and warrants further verification, ideally with more cases.

Among the female population, we found that pattern 3 (DVHC&ILF&MP) was positively associated with the risk of diabetes. This pattern was specifically abundant in the DVHC trajectory for carbohydrate intake, suggesting high levels of dietary carbohydrates as a risk factor for diabetes, and a transition to a moderate level was not effective enough to lower the risk. This finding was consistent with the one from our male population but with a weaker effect. This could be potentially due to the exacerbation of high dietary protein presented in male pattern 2.

To the best of our knowledge, this study was one of the first that utilized a complete observation of macronutrient data in a 21-year cohort to explore the potential heterogeneous trajectory of macronutrient intake. Conventional observational studies measured dietary intake at a single time point, whereas our study captured long-term dietary trends at multiple time points, which may be more suitable for reflecting dietary variations and lifetime macronutrient intake under the occurrence of dietary transition. Compared to those studies that adopted a similar approach and used an incomplete observation with losses to follow-up, our study may be less biased and more accurately explain the trajectories of macronutrients as more data points were included. Nevertheless, this study also broke the routine by expressing the level of macronutrient intake as the absolute intake, which may provide new insights for investigating the relationships between dietary patterns and non-communicable diseases. It is noteworthy that we captured considerable heterogeneity in the trajectory of macronutrient intake between male and female populations, which has not been reported previously in similar studies and may be informative to understand gender differences in long-term dietary intake as well as diet-related behaviors. However, our study also had some limitations. First, the onset of certain diseases may impact on the longitudinal dietary patterns and therefore influence the observed associations. Second, the incidence of cancer, stroke, myocardial infarction, and diabetes was based solely on self-reported information, which may introduce bias into the incidence. Nevertheless, the type of cancer, stroke, and diabetes outcomes were not well categorized as they may exhibit different etiologies. Third, the dietary assessment method used may not be able to accurately reflect the dietary intakes and habits of participants [49]. Last, we did not capture the intakes of different types of macronutrients (e.g., refined carbs versus unrefined carbs, saturated fats versus unsaturated fats, and animal-based proteins versus plant-based protein). Fifth, changes in social living environments over the study period have the potential to distort the relationship between dietary patterns and outcomes; however, this was not fully considered apart from the baseline covariates adjustment conducted in this study. Therefore, we recommended that future studies should take the quality of macronutrients into account when assessing the relationship between dietary intakes and non-communicable diseases.

## 5. Conclusions

In conclusion, our study identified several distinct macronutrient trajectories for both the male and female populations, suggesting that mainland China has undergone dietary transitions during the 20-year period. Our results demonstrated that male pattern 2 (DLC&IHF&MP), characterized by a decreasing low-carbohydrate trajectory and an increasing high-fat trajectory, was associated with a lower risk of all-cause mortality and a higher risk of obesity. Male pattern 3 (DVHC&ILF&UP), characterized by a decreasing very high-carbohydrate trajectory and a U-shape protein trajectory, was associated with a higher risk of diabetes. In addition, male pattern 4 (DHC&ILF&IUP), characterized by a decreasing high-carbohydrate trajectory and an inverted U-shape protein trajectory, was associated with a lower risk of all-cause mortality. Among the female population, our results show that pattern 3 (DVHC&ILF&MP) was positively associated with the risk of diabetes, which was partially consistent with what we found in males. Our findings could potentially provide new insights for epidemiological and nutritional studies that aim to assess the relationship between long-term macronutrient intake and risk of non-communicable diseases.

## Figures and Tables

**Table 1:** Baseline characteristics of the study population by trajectory patterns.

Factors	Male (*n* = 4117)					Female (*n* = 3998)			
	Pattern 1	Pattern 2	Pattern 3	Pattern 4	*p*-Value	Pattern 1	Pattern 2	Pattern 3	*p*-Value
	(*n* = 2223, 54.0%)	(*n* = 1618, 39.3%)	(*n* = 174, 4.2%)	(*n* = 102, 2.5%)		(*n* = 2147, 53.7%)	(*n* = 1619, 40.5%)	(*n* = 232, 5.8%)	
Mean (SD or %)									
Age (years)	30.7 (17.9)	34.2 (15.0)	33.1 (13.0)	29.7 (14.2)	<0.001	35.1 (17.2)	35.4 (13.7)	40.5 (11.5)	<0.001
BMI (kg/m^2^)	19.7 (3.2)	20.8 (3.0)	21.2 (2.4)	20.4 (3.7)	<0.001	20.9 (3.4)	21.7 (3.4)	22.0 (2.7)	<0.001
Weight (kg)	46.5 (18.2)	54.6 (14.9)	57.1 (10.7)	52.9 (16.6)	<0.001	46.2 (14.3)	50.7 (12.1)	52.3 (8.1)	<0.001
Energy Intake (kcal/day)	2522.8 (738.5)	2693.0 (642.7)	3982.4 (979.0)	2865.2 (726.2)	<0.001	2274.6 (566.9)	2411.4 (547.3)	3506.4 (615.9)	<0.001
Carbohydrate Intake (g/day)	439.1 (149.4)	407.7 (109.9)	708.6 (195.5)	505.1 (151.9)	<0.001	394.1 (114.8)	367.3 (98.8)	642.2 (115.2)	<0.001
Fat Intake (g/day)	50.8 (26.2)	79.2 (37.4)	70.3 (50.1)	51.8 (26.7)	<0.001	48.5 (24.0)	73.1 (34.0)	58.2 (38.6)	<0.001
Protein Intake (g/day)	72.1 (22.4)	78.8 (20.6)	124.6 (35.8)	85.3 (26.1)	<0.001	65.1 (17.4)	70.5 (17.8)	103.6 (26.5)	<0.001
CER (%)	69.2 (9.5)	60.9 (9.9)	71.2 (9.9)	70.5 (10.4)	<0.001	69.1 (8.8)	61.1 (9.4)	73.7 (7.9)	<0.001
FER (%)	18.6 (8.9)	26.2 (9.0)	15.9 (9.5)	16.6 (7.9)	<0.001	19.3 (8.5)	27.0 (9.4)	14.5 (7.7)	<0.001
PER (%)	11.5 (1.9)	11.8 (2.1)	12.6 (2.7)	11.9 (1.9)	<0.001	11.5 (1.8)	11.8 (2.1)	11.8 (2.3)	<0.001
Geographic Region									
South	56.5%	73.2%	49.4%	75.5%	<0.001	53.6%	74.0%	45.3%	<0.001
North	43.5%	26.8	50.6%	24.5%		46.4%	26.0%	54.7%	
Location									
Urban	19.9%	45.6%	6.3%	12.7%	<0.001	22.1%	44.7%	6.0%	<0.001
Rural	80.1%	54.4%	93.7%	87.3%		77.9%	55.3%	94.0%	
Education									
Low	64.3%	44.4%	55.7%	51.0%	<0.001	76.5%	57.1%	84.9%	<0.001
Medium	24.4%	33.3%	37.9%	39.2%		16.5%	26.2%	12.9%	
High	11.3%	22.4%	6.3%	9.8%		6.9%	16.7%	2.2%	
Per Capita Household Income									
Low	96.9%	89.7%	97.1%	98.0%	<0.001	95.9%	85.3%	98.3%	<0.001
Medium	2.9%	9.3%	1.7%	1.0%		3.9%	9.6%	0.9%	
High	0.2%	1.0%	1.1%	1.0%		0.1%	1.2%	0.9%	
Marital Status									
In marriage	63.1%	76.9%	70.1%	65.7%	<0.001	78.6%	86.7%	93.1%	<0.001
Other	36.9%	23.1%	29.9%	34.3%		21.4%	13.3%	6.9%	
Alcohol Intake									
None	50.4%	39.6%	41.4%	45.1%	<0.001	89.3%	87.5%	89.7%	NS
<1/week	17.2%	15.6%	25.9%	27.5%		6.2%	7.1%	7.8%	
≥1/week	32.3%	44.8%	32.8%	27.5%		4.4%	5.4%	2.6%	
Smoking									
Never	47.9%	42.8%	35.1%	52.0%	0.002	94.5%	95.9%	96.6%	NS
Former	0.5%	0.4%	0.6%	1.0%		0.0%	0.1%	0.0%	
Current	51.6%	56.8%	64.4%	47.1%		5.5%	4.1%	3.4%	

CER, Carbohydrate-to-energy ratio; FER, fat-to-energy ratio; PER, protein-to-energy ratio; BMI, body mass index; NS: not significant.

## Data Availability

Publicly available datasets were used in this study and can be found at: https://www.cpc.unc.edu/projects/china (accessed on 1 May 2022).

## Supplementary Materials

The following supporting information can be downloaded at: https://www.mdpi.com/article/10.3390/nu16152567/s1, Table S1: Parameters of latent class trajectory models of female participants; Table S2: Parameters of latent class trajectory models of male participants; Table S3: Parameters of latent class analysis for both male and female participants.
