# Supplementary material for: Trajectory Patterns of Macronutrient Intake and Their Associations with Obesity, Diabetes, and All-Cause Mortality: A Longitudinal Analysis over 25 Years
Source: Nutrients. 2024 Aug 5;16(15):2567. doi: 10.3390/nu16152567 (PMC11314385; doi:10.3390/nu16152567)
Supplement: Supplementary file 1 [file nutrients-16-02567-s001.zip › nutrients-3061227-supplementary.pdf]

Table S1. Parameters of latent class trajectory models of female participants.

Model	loglik	Parameters	BIC	Class 1 (%)	Class 2 (%)	Class 3 (%)	Class 4 (%)	Class 5 (%)	Class 6 (%)
Carbohydrate									
1	-189283.3	7	378624.7	100					
2	-188975.8	12	378051.1	24.13	75.86				
3	-188859.5	17	377859.9	22.16	5.33	72.51			
**4**	**-188789.4**	**22**	**377761.3**	**35.74**	**5.08**	**3.87**	**55.3**		
5*	-188857.6	27	377939.1	0	13.71	55.75	11.53	19	
6*	-188753.0	32	377771.5	1.2	2.88	5.17	44.87	34.74	11.16
Fat									
1	-155436.1	7	310930.2	100					
**2**	**-155303.8**	**12**	**310707.1**	**42.4**	**57.5**				
3	-154983.5	17	310107.9	93.14	1.67	5.17			
4	-154878.7	22	309939.8	93.07	1.82	0.52	4.5		
5	-154960.5	27	310144.9	76.4	0.27	4.17	2.52	16.53	
6*	-154767.3	32	309800.0	5	35.6	54.6	0.4	2.52	1.85
Protein									
1	-139821.8	7	279701.7	100					
**2**	**-139673.3**	**12**	**279446.2**	**2.5**	**97.5**				
3	-139542.3	17	279225.6	2.93	1.75	95.3			
4	-139481.2	22	279144.8	94.45	2.67	1.17	1.7		
5*	-139454.1	27	279132.2	51.7	1.42	2.52	5.4	38.9	
6*	-139402.7	32	279070.8	0.18	2.6	5.07	53.3	2.25	36.6

Optimal models were marked in bold. * Model fail to converge.

Table S2. Parameters of latent class trajectory models of male participants

Model	loglik	Parameters	BIC	Class 1 (%)	Class 2 (%)	Class 3 (%)	Class 4 (%)	Class 5 (%)	Class 6 (%)
Carbohydrate									
1	-199164.1	7	398386.4	100					
2	-198860.4	12	397820.6	18.8	81.1				
3	-198667.0	17	397475.5	11.2	21.25	67.47			
4	-198621.7	22	397426.5	10.51	5.14	61.86	22.46		
**5**	**-188857.6**	**27**	**397362.7**	**45.3**	**11.9**	**6.43**	**6.7**	**29.5**	
6*	-198693.4	32	397653.1	23.87	36.82	0.38	0	18.5	20.3
Fat									
1	-162090.1	7	324238.4	100					
2	-161929.9	12	323959.6	53.16	46.83				
**3**	**-161710.5**	**17**	**323562.4**	**4.66**	**37.5**	**57.8**			
4*	-161569.8	22	323322.8	5.24	48.2	7.3	39.2		
5*	-161594.2	27	323413.1	48	0	39.5	5.7	6.65	
6	-161535.3	32	323337.0	2.06	46.6	3.5	11.9	1.19	34.7

Protein									
1	-147980.9	7	296020.0	100					
2	-147832.2	12	295764.2	13.9	86				
**3**	**-147673.0**	**17**	**295487.5**	**5.12**	**90**	**4.8**			
4*	-147651.2	22	295485.5	4.71	12.8	79.6	2.7		
5*	-147578.7	27	295382.2	7.3	4.3	6.55	53.5	28.1	
6*	-147559.1	32	295384.5	56.4	6.3	0.19	5.56	7.35	24.09

Optimal models were marked in bold. * Model fail to converge.

Table S3. Parameters of latent class analysis for both male and female participants.

Model	loglik	Parameters	BIC	AIC
Male				
1	-10506.0461	8	21078.6753	21028.0923
2	-10294.0982	17	20729.6853	20622.1964
3	-10142.4041	26	20501.2030	20336.8081
**4**	**-10077.6702**	**35**	**20446.6411**	**20225.3403**
5	-10070.5690	44	20507.3447	20229.1380
Female				
1	-7081.6254	5	14204.7186	14173.2509
2	-6972.5878	11	14036.4047	13967.1756
**3**	**-6929.2590**	**17**	**13999.5083**	**13892.5180**
4	-6928.6415	23	14048.0347	13903.2830
5	-6928.6283	29	14097.7695	13915.2565

Optimal models were marked in bold.
